# Effect of the Japanese herbal medicine, Boiogito, on the osteoarthritis of the knee with joint effusion

**DOI:** 10.1186/1758-2555-4-3

**Published:** 2012-01-10

**Authors:** Tokifumi Majima, Masahiro Inoue, Yasuhiko Kasahara, Tomohiro Onodera, Daisuke Takahashi, Akio Minami

**Affiliations:** 1 Department of Joint Replacement and Tissue Engineering, Hokkaido University Graduate School of Medicine, Sapporo, Japan; 2 Department of Orthopedic Surgery, Hokkaido University Graduate School of Medicine, Sapporo, Japan

**Keywords:** Osteoarthritis of the knee, Boiogito, Japanese herbal medicine, joint effusion

## Abstract

**Background:**

Boiogito (Japanese herbal medicine, Tsumura Co. Tokyo, Japan) contains *sinomenin *which inhibits inflammatory reactions. Since *sinomenine *is a principle component of the Boiogito, there is a possibility of it being effective on osteoarthritis (OA) of the knee with joint effusion. However, there is no report concerning the effectiveness of Boiogito on knee OA. The objective of the present study is to investigate the therapeutic effect of Boiogito on OA of the knee associated with joint effusion in a comparative study among randomly assigned groups.

**Methods:**

Study was performed using 50 patients who were diagnosed with primary osteoarthritis of the knee with joint effusion. The patients were randomly assigned to two groups: one group (25 patients) using both loxoprofen (2-{4-[(2-oxocyclopentyl) methyl]} propanoic acid) and Boiogito and the other group (25 patients) using loxoprofen, and were evaluated during a 12 week observation period. The assessment parameters including knee scores in the Knee Society Rating System including Knee score and Functional scores, amount of joint effusion by joint puncture in clinically detected cases, the 36-items short form of the Medical Outcome Study Questionnaire (SF-36) as a measurement of health related quality of life were used.

**Results:**

The knee scores based on the Knee Society Rating System were improved in both groups. The staircase climbing up and down ability in the Knee society rating system functional score was significantly improved in the group using Boiogito and loxoprofen compared to the loxoprofen group. In the evaluation using SF-36, significant improvements were found in the scores in both groups in physical functioning after 12 weeks. The amount of joint fluid was significantly decreased at 4, 8 and 12 weeks compared to pre-administration baseline in the group using Boiogito and loxoprofen. A side effect of Boiogito, dry mouth, was found in one case. The symptom was mild and improved immediately after discontinuation of administration.

**Conclusion:**

The results indicated that Boiogito have a possibility for a treatment modality for joint effusion with osteoarthritis of the knee.

## Introduction

Osteoarthritis (OA) of the knee is a degenerative disease of the knee joints which significantly damages the functions of knee joints. The objective of its treatment is to decrease pain while attempting to maintain or increase the range of knee motion and to minimize disabilities in daily living. The majority of osteoarthritic patients visit the clinic with the complaint of pain. As clinical symptoms, joint effusion is frequently associated with pain and limitation in the range of knee motion.

As conservative therapies for OA of the knee, education and physical therapy, oral administration of non-steroidal anti-inflammatory drugs (NSAIDs), intra-articular injection of hyaluronic acid, and use of lateral wedge insole or unloading braces are applied based on the degrees of symptoms [1]. Further, diacerein, which is one kind of herbal medicine, has been used clinically as a symptom modifying drug for reducing joint pain in Europe and the United States of America [2]. It has been reported that diacerein has the chondromodulating effect on the hip joint in observating radiographs among OA patients of the hip joint in a total of 507 cases [3]. Further, diacerein was reported to be effective on OA of the knee [4]. In Asia, the alkaloid, sinomenine is extracted from the Chinese medical plant *Sinomeniuim acutum*, which has been utilized by Chinese doctors for over 2000 years to treat various rheumatic diseases [5]. Previous pharmacological studies have demonstrated that *sinomenine *inhibits inflammatory reactions [5,6].

**Figure 1:**
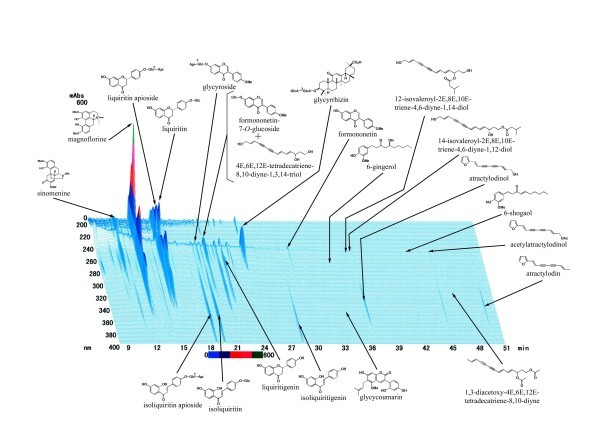
**Three Dimensional pattern of High Performance Liquid Chromatography in Boiogito**. HPLC apparatus consisted of a Shimadzu LC 10A (analysis system software: CLASS-M10A ver. 1.64, Tokyo, Japan) equipped with a multiple wavelength detector (UV 200-400 nm)(Shimadzu SPD-M10AVP, diode array detector), an auto injector (Shimadzu CTO-10AC). HPLC conditions were described as follows: column, ODS (TSK-GEL 80TS, 250 × 4.6 mm i.d., TOSOH, Tokyo, Japan); eluent, (A) 0.05 M AcONH4 (pH 3.6) (B) 100% CH3CN. A linear gradient of 90% A and 10% B changing over 60 min to 0% A and 100% B was used. And 100% B was continued for 20 min.; temperature, 40 degrees celusius; flow rate, 1.0 mL/min. A granule was extracted with methanol (20 mL) under ultrasonication for 30 min., and was centrifuged at 3000 rpm for 5 min. The supernatant was filtrated with a membrane filter (0.45 μm) and then submitted for HPLC analysis (30 μL).

Boiogito (Japanese herbal medicine, Tsumura Co., Tokyo, Japan) contains a dry extract 3.75 g of the mixed drug substance consisting of *Sinomenium Stem *5.0 g, Astragalus Root 5.0 g, Atractylodes Lancea Rhizome 3.0 g, Jujube 3.0 g, Glycyrrhiza 1.5 g, and Ginger 1.0 g in a daily dose of 7.5 g. That is constituted of numerous components including sinomenine as a principal component (Figure 1). Since *sinomenine *is a principle component, there is a possibility of it being effective on OA of the knee with joint effusion. The objective of the present study is to investigate the therapeutic effect of Boiogito on OA of the knee associated with joint effusion in a comparative study among randomly assigned groups.

## Materials and methods

The subjects used were patients with primary OA of the knee associated with clinically detected joint effusion. Those were outpatients having pain in the knee joints while walking and showing a Kellgren-Lawrence [7] grade III or less in the radiographs. The patients having serious complications (liver diseases, kidney diseases, hematological diseases, etc.), those incapable of taking oral administration, those currently taking other herbal medications and those having a hyaluronic acid injection to the joint were excluded from the study. After approval of the Institutional Review Board at the Hokkaido University Hospital, a random, blinded inter-group comparison study was conducted.

The subjects were randomly divided into two groups: one group treated by concomitant use of Boiogito and loxoprofen (thereinafter referred to as the concomitant use group) and loxoprofen administration group (hereinafter referred to as the loxoprofen group). Prior to the study, the subjects signed a written consent of their own free will. The treatment drug, Boiogito, was administered at a daily dose of 7.5 g divided into three portions which were given before meals or between meals. Further, loxoprofen at a single dose of 60 mg was orally administered after meals three times daily. The number of subjects enrolled in the present study was 50 subjects divided into 25 subjects in the concomitant use group and 25 subjects in the loxoprofen group with respect to the treatment drugs. Among them, three cases including two cases (loxoprofen group) who did not come back after the initial examination and one case (concomitant use group) who did not come back after 4^th ^week were excluded from the study. As a result, the remaining 47 subjects (24 cases in the concomitant use group and 23 cases in the loxoprofen group) were used in the analysis of the treatment results.

The period of administration of the treatment drug was 12 weeks. The assessment parameters including knee scores in the Knee Society Rating System including Knee score and Functional scores [8], amount of joint effusion by joint puncture in clinically detected cases, the 36-items short form of the Medical Outcome Study Questionnaire (SF-36) as a measurement of health related quality of life were used. Further, the side effects were investigated.

For statistical analyses, according to the types of data, the following methods were used: χ^2 ^tests for ratio of male and female, Wilcoxon's T- test for patients background, change in SF-36 and Knee Society Rating System knee score and functional scores, one-way analysis of variance for change in volume of joint fluid, and then, a Bonferroni analysis was applied to determine the significance of difference for multiple comparison. Significant level was set at p < 0.05.

## Results

Regarding the patients' background, with respect to gender in both groups, the number of females was greater than the number of males. The mean age was 68.3 years in the concomitant use group and 71.5 years in the loxoprofen group. The detailed preoperative comparison between the 2 groups is shown in Table 1. There were no significant differences in preoperative factors between the two groups.

**Table 1:** Patient demographics

	Concomitant use group (Loxoprofen and Boiogito)	Loxoprofen group (Loxoprofen)	p-value
Number of patients	24	23	-
Sex Female	19	18	-
Male	5	5	-
Average age	68.3 +/- 10.0	71.5 +/- 6.0	N.S
Height (cm)	154.0 +/- 6.9	152.5 +/- 6.2	N.S
Weight (kg)	61.7 +/- 6.2	62.3 +/- 5.7	N.S
Average period of symptoms	14.5 +/- 30	25.0 +/- 23.1	N.S
Knee score in Knee Society Rating System	75.0 +/- 15.1	72.8 +/- 16.0	N.S
Functional score in Knee Society Rating System	32.2 +/- 6.3	28.4 +/- 6.4	N.S
Number of Kellgren-Lawrence Grade	II: 2	II: 2	-
	III:22	III:21	

Data is shown as mean and standard deviation.

The knee score in the Knee Society Rating System was 75.0 +/- 15.1 before administration in the concomitant use group which improved to 85.8 +/- 11.1 at the time of final observation and 72.8 +/- 16.0 before administration in the loxoprofen group which improved to 81.7 +/- 15.1, demonstrating significant improvements in both groups (p < 0.05). The staircase climbing up and down ability in the Knee Society Rating System of functional score significantly improved from 32.2 +/- 6.3 before administration to 40.2 +/- 4.4 at the time of final observation in the concomitant use group (p < 0.05). In the loxoprofen group, the functional score of stairs in Knee Society Rating System changed from 28.4 +/- 6.4 before administration to 36.6 +/- 6.2 at the final follow up. This change has no significant difference.

**Figure 2:**
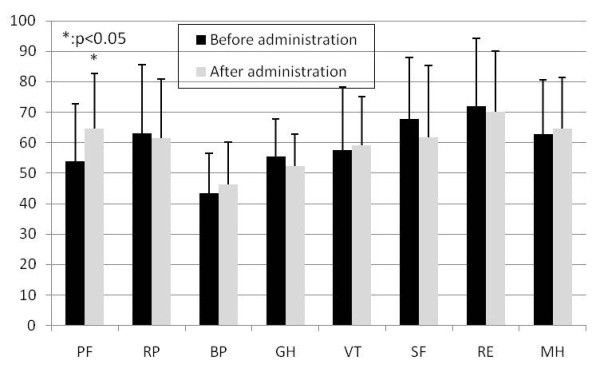
**Change of SF-36 in Boiogito and loxoprofen administration group**. PF: Physical functioning, RP: Role physical, BP: Bodily pain, GH: General health perceptions, VT: Vitality, SF: Social functioning, RE: Role emotion, MH: Mental health. Data is shown as mean and standard deviation.

**Figure 3:**
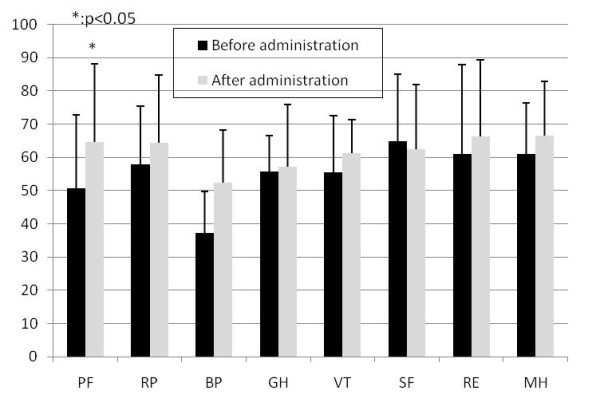
**Change of SF-36 in Loxoprofen administration group**. PF: Physical functioning, RP: Role physical, BP: Bodily pain, GH: General health perceptions, VT: Vitality, SF: Social functioning, RE: Role emotion, MH: Mental health. Data is shown as mean and standard deviation.

In the evaluation of SF-36 which is an assessment of the quality of life of the patients, significant improvements were detected in both groups in the physical functions after 12 weeks (p < 0.05) (Boiogito and loxoprofen administration group in Figure 2, Loxoprofen administration group in Figure 3).

**Figure 4:**
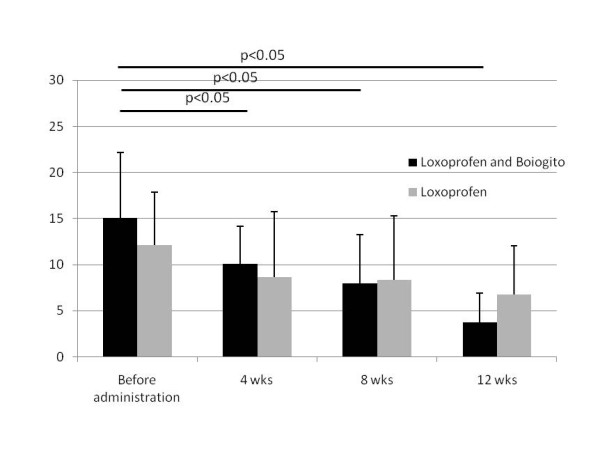
**Change in volume of joint fluid**. Data is shown in mean and standard deviation.

In terms of quantity of joint effusion, the quantity of joint effusion was an average of 15.1 +/- 7.1 ml in the concomitant use group and an average of 12.1 +/- 5.7 ml in the loxoprofen group when punctured before the treatment. There was no significant difference in quantity of joint effusion between two groups before administration. The amounts of joint effusion at the times of 4 weeks, 8 weeks and 12 weeks after treatment were significantly lower compared to those prior to the treatment in the concomitant use group (p < 0.05). On the other hand, there were no significant differences in the amount of joint effusion at the time of 4, 8, and 12 weeks after treatment compared to those before treatment in the loxoprofen group (Figure 4).

As a side effect which seemed to be caused by administration of Boiogito, one case of "dry mouth" was reported. The symptom was mild and was improved immediately after discontinuation of administration.

## Discussion

OA of the knee is a degenerative disease which onsets mostly among the elderly. The number of osteoarthritic patients is simply increasing in society due to the increasing number of the elderly population. The clinical symptoms of OA of the knee include pain as well as limitation in the range of knee motion and joint effusion. As the OA of the knee advances, the symptoms cause a significant effect on activities of daily living.

When the degeneration of knee joints is not severe, conservatinve treatment including drug therapies and physical therapies are selected. For pain management, NSAIDs are widely used. It has been reported that NSAIDs have a major role in the management of OA in a systematic review [9,10]. On the other hand, problems such as an inhibitory action of long-term application of NSAIDs on proteoglycan synthesis of cartilage was pointed out [11,12]. In some cases, joint effusion exists in spite of various treatments. The joint effusion in the knee contains many aggrecan fragments and MMP-1. It has been reported that joint effusion has adverse effects on the articular cartilage [13,14]. According to the results of the present study, joint effusion in OA of the knee significantly decreased by using Boiogito along with NSAIDs. In addition, staircase climbing up and down ability in the knee society score and Physical functioning of SF-36 were found to be improved significantly. These phenomena were assumed to be related to the fact that systematic conditions were improved as a result of improvement in joint effusion by Boiogito. Based on these results, Boiogito was suggested as effective in the conservative treatment for OA of the knee. Boiogito and loxoprofen treatment improve pain and joint effusion, however, their SF-36 scores about another physical area (role physical, bodily pain and general health) were not improve significantly. On the other hand, specific evaluation for knee function according to Knee Society Rating System showed significant difference. These results may be attributed that SF-36 is comprehensive scale and not disease specific scale.

Since herbal medicines consist of numerous components instead of a single component, the mechanisms of decreasing the amount of joint effusion are still unknown. However, with respect to the pharmacological action of Boiogito, effectiveness of *sinomenine *for inflammation which is a component included in the drug substance was reported in the adjuvant arthritis model [15]. It was assumed that *sinomenine *inhibits inflammation of secondary synovitis in OA of the knee. It have been reported that other herbal medicine containing *astragalus *have anti-inflammatory activity through decreasing inflammation related cytokine, Tumor Necrosis Factor (TNF) alpha and Interferon gamma in a rat autoimmune myocarditis model, mice dermatitis model and diabetic mice [16-18]. These reports were not studied on osteoarthritis, however, *astragalus *which is contained in Boiogito have also possibility for decreasing symptoms of osteoarthritis with joint effusion.

Concerning the limitation of the present study, we did not investigate the single administration of Boiogito and also there is a limitation that the number of cases is small. The studies using a larger number of subjects and the studies including analysis of pharmacological actions based on the analysis of the characteristic changes in the joint effusion should be needed. Further, it is necessary to have a long-term assessment including the degree of satisfaction by patients in the general evaluation not only from the local results in the knees.

In conclusion, this is the first report that the herbal remedy "Boiogito" have a possibility of conservative treatment for joint effusion associated with OA of the knee.

## Competing interests

Each author certifies that he has no commercial associations (e.g. consultations, stock ownership, equity interest, patent/licensing arrangements, etc) that might pose a conflict of interest in connection with the submitted article.

## Authors' contributions

TM has made substantial contributions to conception and design, acquisition of data, analysis and interpretation of data. TM, MI, YK, TO and DT carried out the treatment and followed up the patient and contributed to acquisition of data. AM conceived of the study, and participated in its design and coordination. All authors read and approved the final manuscript.
